# Supplementary material for: Discovery and application of insertion-deletion (INDEL) polymorphisms for QTL mapping of early life-history traits in Atlantic salmon
Source: BMC Genomics. 2010 Mar 8;11:156. doi: 10.1186/1471-2164-11-156 (PMC2838853; doi:10.1186/1471-2164-11-156)
Supplement: Additional file 2 — Information on developed 76 locus single-run INDEL panel in Atlantic salmon. Information on fluorescence labeling, primer concentrations, PCR pooling and links to alignments, INDEL motifs and GENESCAN (Burge and Karlin 1997) predictions of genes/exons are available in html format. [file 1471-2164-11-156-S2.ZIP › Additionalfile2/table20411.html]

Contig Alignment

```
Base	A B   
1	C C    
2	T T    
3	A A    
4	G G    
5	T T    
6	T T    
7	A A    
8	G G    
9	A A    
10	T T    
11	T T    
12	A A    
13	G G    
14	T T    
15	T T    
16	C C    
17	T T    
18	T T    
19	T T    
20	A A    
21	T T    
22	T T    
23	C C    
24	A A    
25	C C    
26	T T    
27	C C    
28	A A    
29	A A    
30	T T    
31	T T    
32	C C    
33	A A    
34	C C    
35	G G    
36	T T    
37	C C    
38	T T    
39	G G    
40	A A    
41	A A    
42	A A    
43	T T    
44	A A    
45	A A    
46	C C    
47	A A    
48	T T    
49	T T    
50	C C    
51	C C    
52	A A    
53	C C    
54	A A    
55	C C    
56	A A    
57	T T    
58	A A    
59	C C    
60	A A    
61	T T    
62	A A    
63	C C    
64	A A    
65	T T    
66	C C    
67	C C    
68	T T    
69	A A    
70	A A    
71	T T    
72	T T    
73	A A    
74	G G    
75	A A    
76	C C    
77	T T    
78	G G    
79	A A    
80	A A    
81	C C    
82	A A    
83	A A    
84	A A    
85	A A    
86	A A    
87	T T    
88	A A    
89	T T    
90	A A    
91	A A    
92	A A    
93	C C    
94	G G    
95	C C    
96	A A    
97	A A    
98	C C    
99	A A    
100	C C    
101	A A    
102	A A    
103	C C    
104	A A    
105	A A    
106	T T    
107	T T    
108	T T    
109	T T    
110	A A    
111	C C    
112	T T    
113	G G    
114	A A    
115	A A    
116	T T    
117	T T    
118	A A    
119	C C    
120	A A    
121	G G    
122	T T    
123	T T    
124	C C    
125	A A    
126	T T    
127	A A    
128	G G    
129	A A    
130	A A    
131	G G    
132	G G    
133	A A    
134	A C  1	 transversion  
135	A A    
136	T T    
137	C C    
138	A A    
139	G G    
140	T T    
141	C C    
142	A A    
143	A A    
144	T T    
145	T T    
146	T T    
147	A A    
148	A A    
149	A A    
150	T T    
151	A A    
152	A A    
153	A A    
154	T T    
155	A A    
156	A A    
157	A A    
158	T T    
159	T T    
160	A A    
161	G G    
162	G G    
163	C C    
164	C C    
165	C C    
166	T T    
167	A A    
168	A A    
169	T T    
170	C C    
171	T T    
172	A A    
173	T T    
174	G G    
175	A A    
176	A A    
177	T T    
178	T T    
179	T T    
180	C C    
181	A A    
182	C C    
183	A A    
184	T T    
185	G G    
186	A A    
187	C C    
188	T T    
189	G G    
190	G G    
191	G G    
192	C C    
193	A A    
194	G G    
195	G G    
196	G G    
197	G G    
198	C C    
199	A A    
200	C C    
201	A A    
202	A A    
203	A A    
204	C C    
205	A A    
206	A A    
207	A A    
208	A A    
209	C C    
210	A A    
211	A A    
212	A A    
213	C C    
214	T T    
215	A A    
216	A A    
217	G G    
218	A A    
219	G G    
220	G G    
221	T T    
222	A A    
223	A A    
224	G G    
225	A A    
226	A A    
227	G G    
228	T T    
229	A A    
230	A A    
231	T T    
232	A A    
233	G G    
234	A A    
235	G G    
236	C C    
237	T T    
238	G G    
239	T T    
240	T T    
241	T T    
242	G G    
243	T T    
244	T T    
245	C C    
246	T T    
247	A -  1	 indel  
248	A -  1	 indel  
249	T -  1	 indel  
250	G G    
251	A A    
252	C C    
253	A A    
254	A A    
255	A A    
256	T T    
257	C C    
258	A A    
259	A A    
260	C C    
261	T T    
262	C C    
263	A A    
264	T T    
265	T T    
266	C C    
267	A A    
268	G G    
269	G G    
270	T T    
271	T T    
272	A A    
273	T T    
274	A A    
275	C C    
276	C C    
277	T T    
278	T T    
279	T T    
280	T T    
281	T T    
282	C C    
283	A A    
284	C C    
285	T T    
286	G G    
287	C C    
288	T T    
289	T T    
290	T T    
291	C C    
292	C C    
293	A A    
294	T T    
295	T T    
296	T T    
297	A A    
298	C C    
299	A A    
300	A A    
301	G G    
302	A A    
303	A A    
304	A A    
305	A A    
306	T T    
307	A A    
308	A A    
309	C C    
310	A A    
311	A A    
312	G G    
313	A A    
314	T T    
315	G G    
316	T T    
317	T T    
318	C C    
319	G G    
320	T T    
321	T T    
322	G G    
323	T T    
324	G G    
325	T T    
326	G G    
327	C C    
328	C C    
329	A A    
330	C C    
331	T T    
332	A A    
333	T T    
334	T T    
335	T T    
336	A A    
337	A A    
338	A A    
339	A A    
340	T T    
341	G G    
342	A A    
343	C C    
344	G G    
345	C C    
346	T T    
347	G G    
348	T T    
349	G G    
350	T T    
351	G G    
352	T T    
353	C C    
354	C C    
355	A A    
356	A A    
357	C C    
358	A A    
359	T T    
360	G G    
361	A A    
362	A A    
363	G G    
364	C C    
365	C C    
366	A A    
367	T T    
368	A A    
369	T T    
370	G G    
371	A A    
372	A A    
373	C C    
374	C C    
375	T T    
376	T T    
377	C C    
378	T T    
379	G G    
380	C C    
381	A A    
382	A A    
383	A A    
384	A A    
385	A A    
386	A A    
387	C C    
388	A A    
389	A A    
390	C C    
391	T T    
392	T T    
393	T T    
394	A A    
395	G G    
396	A A    
397	G G    
398	C C    
399	A A    
400	A A    
401	A A    
402	G G    
403	A A    
404	T T    
405	C C    
406	A A    
407	A A    
408	C C    
409	A A    
410	C C    
411	A A    
412	G G    
413	G G    
414	A A    
415	A A    
416	G G    
417	T T    
418	A A    
419	G G    
420	T T    
421	G G    
422	A A    
423	A A    
424	A A    
425	C C    
426	A A    
427	A A    
428	T T    
429	A A    
430	A A    
431	C C    
432	A A    
433	G G    
434	A A    
435	A A    
436	C C    
437	A A    
438	T T    
439	C C    
440	T T    
441	G G    
442	A A    
443	C C    
444	T T    
445	G G    
446	A A    
447	A A    
448	G G    
449	G G    
450	T T    
451	T T    
452	A A    
453	C C    
454	A A    
455	G G    
456	A A    
457	G G    
458	C C    
459	T T    
460	C C    
461	A A    
462	A A    
463	T T    
464	A A    
465	C C    
466	A A    
467	G G    
468	G G    
469	A A    
470	A A    
471	G G    
472	T T    
473	A A    
474	G G    
475	T T    
476	G G    
477	A A    
478	A A    
479	A A    
480	C C    
481	A A    
482	A A    
483	T T    
484	A A    
485	A A    
486	C C    
487	A A    
488	G G    
489	A A    
490	A A    
491	C C    
492	A A    
493	T T    
494	C C    
495	T T    
496	G G    
497	A A    
498	C C    
499	T T    
500	G G    
501	A A    
502	A A    
503	G G    
504	G G    
505	T T    
506	T T    
507	A A    
508	C C    
509	A A    
510	G G    
511	A A    
512	G G    
513	A A    
514	T T    
515	C C    
516	A A    
517	C C    
518	A A    
519	C C    
520	T T    
521	A A    
522	T T    
523	A A    
524	T T    
525	A A    
526	A A    
527	A A    
528	G G    
529	A A    
530	G C  1	 transversion  
531	. A    
532	. G    
533	. A    
534	. G    
535	. G    
536	. C    
537	. T    
538	. G    
539	. T    
540	. C    
541	. A    
542	. C    
543	. C    
544	. C    
545	. T    
546	. A    
547	. A    
548	. T    
549	. C    
550	. C    
551	. C    
552	. T    
553	. A    
554	. C    
555	. C    
556	. T    
557	. A    
558	. G    
559	. T    
560	. G    
561	. C    
562	. A    
563	. C    
564	. T    
565	. A    
566	. C    
567	. C    
568	. T    
569	. T    
570	. C    
571	. A    
572	. G    
573	. G    
574	. C    
575	. T    
576	. C    
577	. T    
578	. T    
579	. G    
580	. T    
581	. A    
582	. G    
583	. T    
584	. G    
585	. C    
586	. A    
587	. C    
588	. T    
589	. A    
590	. T    
591	. G    
592	. T    
593	. A    
594	. G    
595	. G    
596	. G    
597	. A    
598	. A    
599	. T    
600	. A    
601	. G    
602	. G    
603	. G    
604	. T    
605	. G    
606	. C    
607	. C    
608	. A    
609	. T    
610	. T    
611	. T    
612	. G    
613	. T    
614	. G    
615	. A    
616	. C    
617	. A    
618	. G    
619	. T    
620	. C    
621	. T    
622	. A    
623	. A    
624	. G    
625	. A    
626	. G    
627	. A    
628	. A    
629	. T    
630	. T    
631	. A    
632	. A    
633	. C    
634	. G    
635	. T    
636	. A    
637	. A    
638	. T    
639	. G    
640	. A    
641	. A    
642	. G    
643	. T    
644	. A    
645	. T    
646	. G    
647	. A    
648	. T    
649	. G    
650	. T    
651	. C    
652	. A    
653	. A    
654	. C    
655	. A    
656	. A    
657	. A    
658	. G    
659	. G    
660	. C    
661	. C    
662	. C    
663	. T    
664	. T    
665	. G    
666	. T    
667	. T    
668	. T    
669	. C    
670	. C    
671	. T    
672	. C    
673	. T    
674	. T    
675	. C    
676	. T    
677	. C    
678	. A    
679	. C    
680	. C    
681	. G    
682	. T    
683	. C    
684	. A    
685	. T    
686	. C    
687	. C    
688	. T    
689	. A    
690	. A    
691	. A    
692	. A    
693	. A    
694	. C    
695	. C    
696	. T    
697	. C    
698	. C    
699	. A    
700	. T    
701	. A    
702	. A    
703	. C    
704	. A    
705	. T    
706	. C    
707	. T    
708	. A    
709	. G    
710	. A    
711	. A    
712	. A    
713	. T    
714	. A    
715	. A    
716	. T    
717	. G    
718	. A    
719	. T    
720	. A    
721	. T    
722	. T    
723	. A    
724	. T    
725	. T    
726	. T    
727	. G    
728	. A    
729	. T    
730	. C    
731	. A    
732	. A    
733	. A    
734	. T    
735	. G    
736	. T    
737	. G    
738	. T    
739	. A    
740	. A    
741	. C    
742	. T    
743	. T    
744	. C    
745	. G    
746	. T    
747	. G    
748	. C    
749	. A    
750	. G    
751	. A    
752	. G    
753	. A    
754	. A    
755	. A    
756	. C    
757	. A    
758	. A    
759	. C    
760	. A    
761	. C    
762	. A    
763	. G    
764	. G    
765	. T    
766	. C    
767	. T    
768	. A    
769	. G    
770	. A    
771	. C    
772	. A    
773	. G    
774	. C    
775	. G    
776	. A    
777	. C    
778	. T    
779	. C    
780	. C    
781	. T    
782	. T    
783	. T    
784	. T    
785	. A    
786	. T    
787	. T    
788	. T    
789	. A    
790	. A    
791	. C    
792	. T    
793	. T    
794	. T    
795	. A    
796	. T    
797	. A    
798	. T    
799	. T    
800	. A    
801	. A    
802	. T    
803	. T    
804	. A    
805	. T    
806	. C    
807	. A    
808	. T    
809	. C    
810	. A    
811	. G    
812	. A    
813	. G    
814	. T    
815	. C    
816	. C    
817	. A    
818	. T    
819	. T    
820	. G    
821	. C    
822	. A    
823	. C    
824	. A    
825	. G    
826	. C    
827	. T    
828	. G    
829	. T    
830	. G    
831	. T    
832	. C    
833	. A    
834	. G    
835	. T    
836	. C    
837	. A
```
